# Standardized Human Platelet Lysates as Adequate Substitute to Fetal Calf Serum in Endothelial Cell Culture for Tissue Engineering

**DOI:** 10.1155/2022/3807314

**Published:** 2022-03-03

**Authors:** Katharina Peters, Tania Helmert, Susanne Gebhard, Volker Mailänder, Ronald E. Unger, Sandra Nezi-Cahn, Annette Hasenburg, Martin Heller, Roxana Schwab, Walburgis Brenner

**Affiliations:** ^1^Department of Obstetrics and Gynecology, University Medical Center of the Johannes Gutenberg University Mainz, Mainz, Germany; ^2^BiomaTiCS-Biomaterials, Tissues, and Cells in Science, University Medical Center of the Johannes Gutenberg University Mainz, Mainz, Germany; ^3^Max Planck Institute for Polymer Research, Mainz, Germany; ^4^Department of Dermatology and Venerology, University Medical Center of the Johannes Gutenberg University Mainz, Mainz, Germany; ^5^Institute of Pathology, University Medical Center of the Johannes Gutenberg University Mainz, Mainz, Germany

## Abstract

Fetal calf serum (FCS) is used for *in vitro* cell culture, as it provides the cells with various growth-promoting compounds. For applications in humans, FCS does not meet the required safety standards and should be replaced by an appropriate substitute. This study analyzed the suitability of using human platelet lysate (hPL) as a substitute for FCS in endothelial cell cultures for *in vitro* and *in vivo* tissue engineering applications. The focus was placed on standardized, commercially available hPLs (MultiPL'30, MultiPL'100), which are approved for applications in humans, and compared to laboratory-prepared hPLs (lp-hLP). Human umbilical vein endothelial cells (HUVEC) were cultured with FCS or with different hPLs. Cell morphology, proliferation, viability, apoptosis, and necrosis, as well as the organization of vascular structures, were assessed. No morphological changes were noticed when FCS was replaced by standardized hPLs in concentrations of 1-10%. In contrast, the use of lp-hLPs led to irregular cell shape and increased vacuolization of the cytoplasm. HUVEC proliferation and viability were not compromised by using media supplemented with standardized hPLs or pl-hPLs in concentrations of 1-10%, compared to cells grown in media supplemented with 20% FCS. The apoptosis rate using lp-hPLs was higher compared to the use of standardized hPLs. The necrosis rate tended to be lower when FCS was replaced by hPLs. HUVEC formed more pronounced capillary-like structures when the media were supplemented with hPLs instead of supplementation with FCS. Thus, compared to the use of FCS, the use of hPLs was beneficial for the growth and optimal expression of functional endothelial cell characteristics during *in vitro* experiments. Commercially available hPLs proved to be particularly suitable, as they led to reproducible results during *in vitro* experiments, while meeting the safety requirements for in vivo use.

## 1. Introduction

Tissue engineering is of increasing importance in reconstructive medicine as it provides functional grafts for replacing damaged or missing tissues and organs [1–3]. Tissue engineering can be used in various applications, including skin substitutes for wound closure in chronic wounds, trauma deformities, or after gyneco-oncological or uro-oncological procedures with extensive tissue defects [3–5], such as vulvar or vaginal cancer. Nevertheless, the transition from bench to bedside is a challenging and time-consuming process, which requires a thorough balance of functionality and efficacy of the tissues provided on one side, and the safety of the recipient on the other [1].

Insufficient vascularization is the limiting factor in preclinical and clinical applications of in vitro engineered tissues [6]. To improve the chances of graft survival during i*in vivo* applications, it is crucial that the engineered grafts are already prevascularized, and that additional vascular structures are rapidly formed within the engineered tissues [7, 8]. For the purpose of prevascularization, endothelial cells were already used during the *in vitro* tissue generation processes [9–11]. Prevascularized tissue constructs were successfully tested in animal models and provided a fast anastomosis with the recipient's blood vessel system [10, 12]. To date, several sources of cells of endothelial origin, for example human umbilical vein endothelial cells (HUVEC), proved to be suitable for the generation of *in vitro* prevascularized engineered tissues [10, 13–15]. For clinical applications, tissue engineered products should fulfill strict safety criteria for applications in humans [16].

Fetal calf serum (FCS) is widely used as a supplement in cell culture media [15, 17, 18]. FCS supplies the cells with, i.a., nutrients and growth factors to generate an optimal microenvironment for cell cultivation, thus assuring appropriate cell viability, proliferation, and functionality [17, 19, 20]. However, the exact composition of FCS is unknown and varies from batch to batch [21]. For safety reasons, the use of preparations or solutions in humans is prohibited, if they contain unknown nutrients or proteins or their concentration is not clearly defined [21]. Furthermore, the utilization of FCS in humans may expose the recipients to an infection threat, as it may contain impurities or infectious particles, such as animal viruses or prions [22, 23]. Moreover, substantial ethical considerations are linked to the manufacturing process of FCS, as it is extracted from the blood of unborn calf fetuses, and as a result, up to two million animals are sacrificed annually [24, 25]. Thus, to comply with safety regulations and ethical standards, FCS should be replaced by an adequate substitute for in vitro cell culture and for the tissue engineering process, if the cells or tissues are aimed to be used for applications in humans.

FCS-free media, supplemented with human alternative products, were recently described for several applications. A possible substitute for FCS is the use of human platelet lysates (hPLs) obtained from human platelet-rich blood plasma [26]. Platelets contain alpha granules carrying various growth factors, which are crucial for cell viability and cell proliferation [27–29]. However, the exact composition of the respective hPL may vary from donor to donor [20]. Commercially available pooled lysates may trigger immunological reactions [30]; thus, in order to implement tissue engineered products into clinical practice, it is absolutely necessary to use a standardized, commercially available supplement as substitute for FCS. Previous studies showed that the supplementation of cell growth media with hPL instead of FCS for the cultivation of bone marrow-derived mesenchymal stromal cells did not negatively affected cell behavior and functions [30, 31]. The use of media supplemented with 5% laboratory-prepared hPL from pooled buffy coats showed no significant negative effects on the cell metabolic activity of various types of endothelial cells compared to the use of media supplemented with 5% FCS [31]. Thus, hPL is an appropriate FCS substitute which was already tested for the use in humans. The use of standardized, commercially available hPLs could be advantageous to avoid variations in growth factors during the cultivation of endothelial cells.

This study is aimed at analyzing if it is feasible to use hPLs instead of FCS for tissue engineering purposes, especially regarding the cultivation of endothelial cells and the formation of vascular structures during in vivo cell culture. For this purpose, the endothelial cell culture medium was supplemented with either two commercially available, standardized hPL solutions or with a laboratory-prepared hPL (lp-hPL) solution. Endothelial cell growth, proliferation, and cell function were evaluated in cells grown in media supplemented with the three different lysates.

## 2. Materials and Methods

### 2.1. Platelet Lysates

Two standardized, commercially available and approved hPLs, MultiPL'30 and MultiPL'100, were obtained from Maco Pharma International GmbH (Langen, Germany). MultiPL'30 consists of 30% plasma and 70% additive solution and is produced from a buffy coat. MultiPL'100 consists of 100% plasma and is produced through apheresis donation.

A laboratory-prepared hPL was generated from human apheresis platelet concentrates, as described by a previously published, standardized freeze-thaw method [32]. The human platelet apheresis products from ten healthy, anonymous donors were provided by the Transfusion Center of the University Medical Center Mainz in accordance with the Declaration of Helsinki (Approval of the local Ethics Committee, No. 837.439.12). The content of the bags was pooled, aliquoted, frozen for 10 to 40 min at -80°C, and then thawed for 7.5 min at 37°C in a water bath for five cycles. To deplete cellular debris, the laboratory-prepared platelet lysate was centrifuged at 1410 g for 40 min at 22°C (5702R, Eppendorf, Germany). The supernatant was collected, aliquoted, and stored at -80°C until used. This platelet lysate contained residual plasma.

### 2.2. HUVEC Isolation and Cultivation

Freshly delivered umbilical cords were kindly provided by the Clinic of Obstetrics and Women's Health of the University Medical Center Mainz. Umbilical cords were obtained anonymously after informed consent of the patients in accordance with the Declaration of Helsinki. The inner lining of the umbilical cord vein was incubated with collagenase II (Biochrom, Germany) solution (1 mg/ml) at 37°C and 5% CO_2_ for 15 min and finally rinsed with cell culture medium (medium 199 supplemented with 25 *μ*g/ml GlutaMAX, 25 *μ*g/ml endothelial cell growth supplement (Sigma-Aldrich, USA), 100 U/100 *μ*g/ml penicillin/streptomycin (Thermo-Fisher, USA), and 20% FCS (Gibco, USA)). The suspended HUVEC were centrifuged, resuspended in cell culture medium, and transferred to cell culture flasks (75 cm^2^, Greiner Bio-One, Germany). HUVEC identity was tested and verified by PECAM-1 staining [33]. The isolated cells were 100% positive for PECAM-1 (data not shown). Cells were isolated from the umbilical cords of three donors. HUVEC from the three different donors were used for the three independent experimental runs. Cells were used in the first to third passage.

The HUVEC cells were processed strictly anonymously without recording patient-related data and in accordance with local ethical regulations (Approval of the local Ethics Committee, No. 15794_1). The research was carried out in accordance with the World Medical Association Declaration of Helsinki, and all subjects provided written informed consent.

### 2.3. Light Microscopic Examination

For light microscopy, lower cell numbers were used than for cell functional assays in order to assess cell morphology properly. HUVEC were seeded in a 12-well plate with a density of 4 × 10^4^ cells per well. Morphology was assessed (VHX-1000, Keyence, Japan) after 72 h cellular growth in culture media supplemented with either 20% FCS or with 1%-10% of the various hPLs, respectively.

### 2.4. Cell Proliferation Assay

Cell proliferation was assessed by BrdU (Bromodeoxyuridine) incorporation into replicating DNA. HUVEC were seeded in a 96-well plate, coated with 0.2% gelatin at a density of 5 × 10^3^ cells per well, and grown in 200 *μ*l culture medium at 37°C and 5% CO_2_ for 24 h. The cell density was used according to the specification of the assay kit. Afterwards, the culture medium was removed and replaced with 200 *μ*l fresh medium supplemented with either 20% FCS or with different concentrations (1%-10%) of the various hPLs. After 72 h, HUVEC were labeled with BrdU (incubation time 2 h) and analyzed using a Cell Proliferation ELISA Kit (Sigma-Aldrich, USA).

### 2.5. Cell Viability Assay

Cell viability was assessed by the MTT assay. HUVEC were seeded in a 96-well plate coated with 0.2% gelatin (Sigma-Aldrich, USA) at a density of 5 × 10^3^ cells per well and cultured in 100 *μ*l cell culture medium at 37°C and 5% CO_2_. The cell density was used according to the specification of the assay kit. After 24 h, the culture medium was replaced with 100 *μ*l fresh medium supplemented with either 20% FCS or with different concentrations (1%-10%) of the various hPLs, and cultivation was continued for an additional 72 h. For the MTT assay, HUVEC were incubated in 20 *μ*l of 0.5 mg/ml MTT (Sigma-Aldrich, USA) at 37°C and 5% CO_2_ for 4 h. Then, the solution was removed, and 100 *μ*l isopropyl alcohol (AppliChem, Germany) was added for 15 min, accompanied by vigorous shaking. Absorbance was measured at 570 nm (reference wavelength: 650 nm) with a microplate reader (GloMax® Microplate Reader, Promega, Germany).

### 2.6. Apoptosis and Necrosis Assay

Apoptosis and necrosis were analyzed by RealTime-Glo™ Annexin V Apoptosis and Necrosis Assay Kit (Promega, Germany). This assay combined positive luminescence for apoptosis detection (exposure of phosphatidylserine (PS) to the outer leaflet of the cell membrane) and additional positive fluorescence (DNA exposure by loss of membrane integrity) for necrosis detection.

Four days prior to stimulation, HUVEC were seeded at a density of 5 × 10^3^ cells per well in a 96-well plate (used cell density according to the specification of the assay kit), coated with 0.2% gelatin and grown in culture medium at 37°C and 5% CO_2_ for 24 h. Afterwards, the culture medium was replaced by 200 *μ*l fresh medium supplemented with either 20% FCS or with the various concentrations (1%-10%) of different hPLs, and incubation was continued for 72 h. Annexin-V staining was accomplished following the manufacturer's instructions.

### 2.7. Tube Formation Assay

The ability of the endothelial cells to organize themselves into vascular structures was assessed by the tube formation assay. After polymerizing 15 *μ*l Matrigel (BD Biosciences, USA) on *μ*-slides (ibidi, Germany) at 37°C and 5% CO_2_ for 30 min, the gel was overlaid with 9 × 10^3^ HUVEC in 50 *μ*l per well. The cells were previously grown in media supplemented with either 20% FCS or with various concentrations (1%-10%) of different hPLs for a period of 24 h. Tube formation was assessed by addition of 1 *μ*l calcein-AM (Thermo Fisher, USA) to each well 4.5 h after seeding the cells. Tube formation was documented by fluorescence microscopy (Leica, Germany). The quantitative evaluation of tube length and branching points resulted in erroneous results due to the partially confluent arrangement of cells and the uneven distribution of tubes in the single preparations. We have therefore opted for a purely qualitative evaluation.

### 2.8. Statistical Analysis

All experiments were conducted in triplicate (proliferation and viability assays were performed in a 4-fold approach, apoptosis, and necrosis assays, as well as tube formation assays were performed in a 2-fold approach). Statistical analysis was performed with Microsoft Excel. Significant differences were calculated using the two-sided Student *t*-test and represented as mean values ± standard error of the mean (S.E.M.). The level of statistical significance was set at *p* < 0.05.

## 3. Results

### 3.1. Morphological Assessment by Light Microscopy

**Figure 1:**
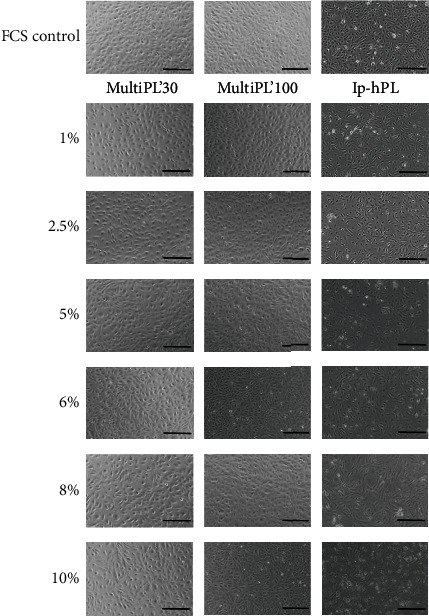
Morphology of HUVEC grown in medium supplemented with either 20% fetal calf serum (FCS) or with different concentrations of human platelet lysates. HUVEC were seeded in a 12-well plate at a density of 4 × 10^4^ cells/well and grown for 72 h in either cell culture media supplemented with either 20% FCS or with human platelet lysates MultiPL'30, MultiPL'100, or with laboratory-prepared human platelet lysate (lp-hPL) in different concentrations. Representative light microscopy images of three independent experiments are shown. Scale bars represent 200 *μ*m.

No morphological changes in HUVEC morphology were observed after cell culture in media supplemented with either MultiPL'30 or MultiPL'100, independent of their respective concentrations and compared to the control group (HUVEC grown in culture medium supplemented with 20% FCS) (Figure 1). However, HUVEC grown in medium supplemented with lp-hPL showed an altered morphology with irregular shape and increased vacuolization of the cytoplasm, which was more pronounced with growing lp-hPL concentrations (Figure 1).

### 3.2. Cell Proliferation

**Figure 2:**
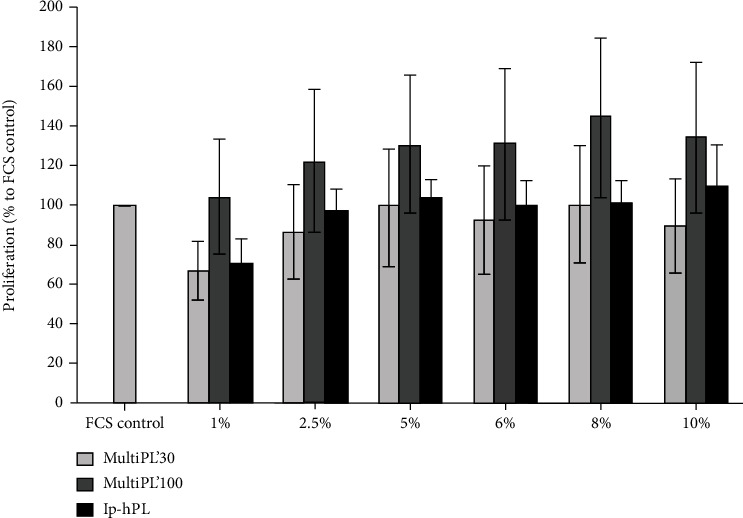
Proliferation of HUVEC cultured in media supplemented with either fetal calf serum (FCS) or with human platelet lysates. HUVEC were seeded in a 96-well plate at a density of 5 × 10^3^ cells/well and grown for 72 h in media supplemented with either 20% FCS (controls) or with standardized, commercial human platelet lysates MultiPL'30, MultiPL'100, or with laboratory-prepared human platelet lysate (lp-hPL) at different concentrations. Proliferation was determined by BrdU assay and is shown as percentage of HUVEC grown in medium supplemented with 20% FCS (control). Data are expressed as mean ± S.E.M. Experiments were conducted in triplicate and repeated three times.

HUVEC cultured in medium supplemented with 1% MultiPL'30 showed a reduced proliferation (33%, no statistical significance), compared to the control group (cells grown in medium supplemented with 20% FCS) (Figure 2). Proliferation of cells cultivated in medium supplemented with MultiPL'30 in higher concentrations (up to 10%) was similar to the proliferation in the control group (cells cultured in medium supplemented with FCS). HUVEC cultivation in medium supplemented with MultiPL'100 resulted in a concentration-dependent increased proliferation compared to the controls, but without reaching statistical significance (Figure 2). Cultivation of HUVEC in medium supplemented with lp-hPL led to a slightly decreased proliferation (28%) at a concentration of 1% lp-hPL compared to controls, but without reaching statistical significance. HUVEC cultured in medium with lp-hPL in higher concentrations of up to 10% showed proliferation rates similar to the control group (cells cultured in medium supplemented with FCS) (Figure 2).

### 3.3. Cell Viability

**Figure 3:**
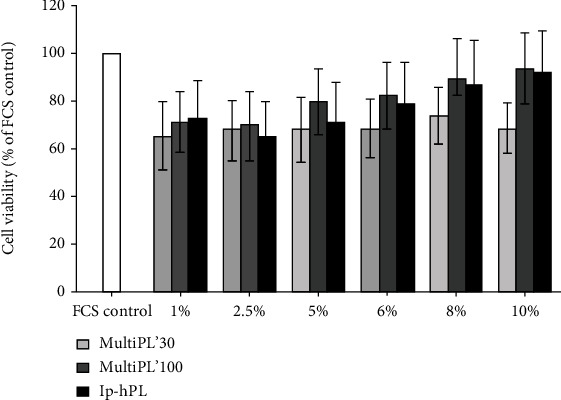
Cell viability of HUVEC cultured in media supplemented with either fetal calf serum (FCS) or with human platelet lysates. HUVEC were seeded in a 96-well plate at a density of 5 × 10^3^ cells/well and grown for 72 h in media supplemented with either 20% FCS (controls) or with standardized, commercial human platelet lysates MultiPL'30, MultiPL'100, or laboratory-prepared human platelet lysate (lp-hPL) at different concentrations. Cell viability was determined by the MTT assay. Data are expressed as mean ± S.E.M. Experiments were conducted in triplicate and repeated three times.

Cell viability was assessed by the MTT-assay. HUVEC cultured in media supplemented with 1% to 10% MultiPL'30 showed a decreased cell viability of approx. 30% compared to cells cultured in medium supplemented with 20% FCS, but this reached no statistical significance (Figure 3). Cell viability of HUVEC grown in media supplemented with MultiPL'100 or with lp-hPL (concentrations of 1% to 8%) also showed a nonsignificant decrease in cell viability, but a similar cell viability to controls, when cultured in media supplemented with the respective platelet lysates in concentrations of 10% compared to the viability of cells grown in 20% FCS (Figure 3).

### 3.4. Cell Apoptosis

**Figure 4:**
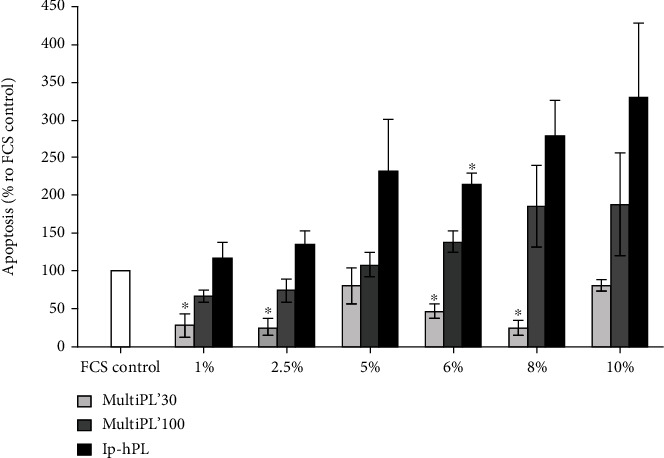
Apoptosis of HUVEC cultured in media supplemented with either fetal calf serum (FCS) or with human platelet lysates. Cell viability was determined by RealTime-Glo™ Annexin V Apoptosis and Necrosis Assay. HUVEC were seeded in a 96-well plate at a density of 5 × 10^3^ cells/well and grown for 72 h in media supplemented with either 20% FCS (controls) or with standardized, commercial-available human platelet lysates MultiPL'30, MultiPL'100, or laboratory-prepared human platelet lysate (lp-hPL) at different concentrations. Data are expressed as mean ± S.E.M. Experiments were conducted in duplicates and repeated three times. ^∗^ indicates *p* < 0.05 (Student's *t*-test).

The supplementation of cell growth media with 1%, 2.5%, 6%, and 8% MultiPL'30 led to significantly reduced apoptosis of HUVEC compared to controls (cells grown in medium supplemented with 20% FCS). The supplementation of cell growth medium with 10% MultiPL'30 resulted in a similar apoptosis rate as shown by controls (cells grown in medium supplemented in 20% FCS) (Figure 4). In contrast, the replacement of FCS with MultiPL'100 or lp-hPL showed a trend to increased HUVEC apoptosis (Figure 4). This phenomenon was more pronounced when the medium was supplemented with platelet lysates in higher concentrations. The medium supplementation with 6% lp-hPL even led to a significantly increased apoptosis rate compared to controls (Figure 4).

### 3.5. Cell Necrosis

**Figure 5:**
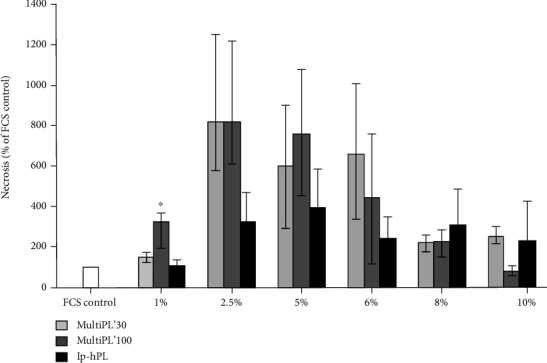
Necrosis of HUVEC cultured in media supplemented with either fetal calf serum (FCS) or with human platelet lysates. Cell viability was determined by RealTime-Glo™ Annexin V Apoptosis and Necrosis Assay. HUVEC were seeded in a 96-well plate at a density of 5 × 10^3^ cells/well and grown for 72 h in media supplemented with either 20% FCS (controls) or with standardized, commercial human platelet lysates MultiPL'30, MultiPL'100, or with laboratory-prepared human platelet lysate (lp-hPL) at different concentrations. Data are expressed as mean ± S.E.M. Experiments were conducted in duplicates and repeated three times. ^∗^ indicates *p* < 0.05 (Student's *t*-test).

Cell necrosis was assessed by RealTime-Glo™ Annexin V Apoptosis and Necrosis Assay Kit after cell growth medium was supplemented with the different hPLs. Cells grown in media supplemented with concentrations of 2.5% to 8% of all three hPLs showed a trend toward increased necrosis, with a maximum necrosis rate in cells grown in media supplemented with 2.5% to 5% hPL, compared to controls (cells grown in medium supplemented with 20% FCS). However, the necrosis rate of cells grown in medium supplemented with 10% of hPLs was comparable to the necrosis rate of cells seeded in medium supplemented with 20% FCS (Figure 5). Cells seeded in medium supplemented with 1% MultiPL'100 displayed significantly increased necrosis compared to controls (Figure 5).

### 3.6. Tube Formation

**Figure 6:**
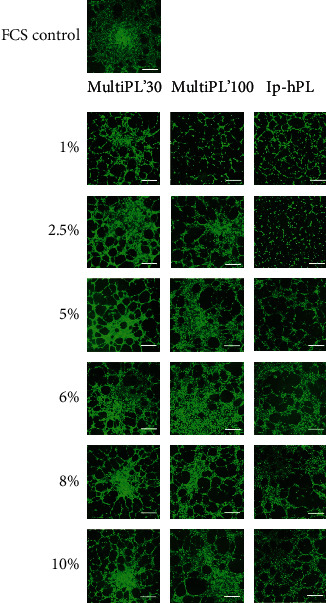
Tube formation of HUVEC cultivated in media supplemented with either fetal calf serum (FCS) or with human platelet lysates in different concentrations. HUVEC were seeded on *μ*-slides with a density of 9 × 10^3^ cells/well after the cells were grown for 24 h in culture media supplemented with either 20% FCS or with MultiPL'30, MultiPL'100, or with laboratory-prepared human platelet lysate (lp-hPL). Images of tube-like structures were taken after 4.5 h with fluorescence microscopy after calcein-AM staining. Representative light microscopy images of three independent experiments are shown. Scale bars represent 200 *μ*m.

Cultivation of HUVEC in media supplemented with hPLs resulted in a more pronounced formation of capillary-like structures than with cultivation of endothelial cells in medium supplemented with FCS. The most pronounced tube formation was observed when using MultiPL'30 with an optimum at a concentration of 5%. During cultivation in media supplemented with MultiPL'100, the most pronounced tube formation was observed at a concentration of 8% and with lp-hPL at 10% platelet lysate (Figure 6).

## 4. Discussion

Tissue engineering of grafts that are functional and safe for recipients is of great importance in regenerative medicine. The supplementation of medium with FCS for the cultivation of primary endothelial cells in order to engineer *in vitro* tissues has been indispensable. Thus, the replacement of FCS with a suitable growth substitute, that is equally effective and assures cell health and functioning, is a prerequisite to develop tissue engineering products for the use in clinical applications. In this study, we showed that primary HUVEC, cultivated in cell culture media supplemented with hPL, showed no functional loss compared to cells grown in medium supplemented with FCS. Additionally, cell behavior of cells grown in media supplemented with 1% to 10% of hPLs was similar to cell behavior of the controls (cells grown in medium supplemented with the standard concentration of 20% FCS). Especially with regard to the formation of capillary-like structures, as prerequisite for the engineering of prevascularized tissues, the use of cell growth media supplemented with commercially available hPLs proved to be clearly beneficial compared to cell growth media supplemented with laboratory-prepared hPL.

Growth factors are crucial for regulation of essential cell functions, as they activate downstream cascades which orchestrate cell proliferation, cell survival, and cellular growth. Previous studies showed that human platelet lysates contain a range of growth factors which are similar to FCS [17, 20, 34]. Different compositions and concentrations of growth factors influence cell functionality and cell behavior [21]. In FCS, as well as in nonstandardized hPL, the range of growth factors is very broad [17, 20, 35] and is not examined in routine applications. The impairment of cell functionality and cell behavior can manifest itself in altered cell morphology [36]. The morphology of HUVEC did not differ between cells cultivated in media supplemented with 20% FCS and cells grown in media supplemented with the commercially available human platelet lysates MultiPL'30 and MultiPL'100 in concentrations of 1% to 10%. HUVEC retained their typical cobblestone morphology and exhibited no signs of cellular stress, such as vacuolization of the cytoplasm. Thus, the concentrations of various factors within the commercially available hPLs did not negatively impact HUVEC cell functionality and cell behavior. In contrast, when the growth medium was supplemented with lp-hPL, HUVEC showed irregular cell shape and increased vacuolization of the cytoplasm, presumably as a result of fluctuations of the composition of growth factors.

The commercially available hPLs contain, i.a., insulin-like growth factor-1 (IGF-1), vascular endothelial growth factor (VEGF), and epidermal growth factor (EGF) (Maco Pharma International GmbH, MultiPL'30 and MultiPL'100 information sheets; 2017). Cell proliferation and cell viability of HUVEC were not significantly affected when replacing FCS in culture media with any of the three tested hPLs in various concentrations. The supplementation of the media with up to 10% hPLs led to a comparable proliferation rate and cell viability as observed in controls (cells grown in medium supplemented with 20% FCS). Additionally, cell proliferation and cell viability increased in a dose-dependent manner with rising concentrations of hPLs, presumably as a result of increased amount of growth factors. VEGF plays an important role in cell proliferation and cell viability and is a key player in angiogenesis [37, 38]. The binding of VEGF to VEGF-R activates various metabolic pathways, such as the RAS/RAF/MEK/ERK cascade. Activated ERK activates transcription factors that influence endothelial cell proliferation and survival [37]. While VEGF concentration in the commercially available MultiPL'30 is approx. 900 pg/ml and in MultiPL'100 approx. 600 pg/ml (Maco Pharma International GmbH, MultiPL'30 and MultiPL'100 information sheets; 2017), those in noncommercially available platelet lysates vary between 150 pg/ml and 7000 pg/ml. Moreover, the concentration of VEGF in FCS is substantially lower (2.5 pg/ml) [17, 20, 35]. We hypothesize that the higher concentration of VEGF in the hPLs explains the fact that supplementation with low concentrations of hPL was sufficient to achieve a comparable proliferation rate and cell viability as in controls (cells grown in media supplemented with 20% FCS).

The growth factor EGF is a mitogen that also promotes the proliferation of endothelial cells [39]. The binding of EGF to its receptor activates various metabolic pathways responsible for cellular growth and proliferation, such as RAS/RAF/MEK/ERK, JAK/STAT, PI3K/AKT/mTOR, and PLC*γ*/PKC [40]. FCS contains betacellulin, which belongs to the EGF family and binds to the EGF receptor as well [41]. Human platelet lysates and FCS have a similar concentration range of EGF. FCS contains approximately 3.7 ng/ml of betacellulin [41], and EGF concentrations of the commercially available platelet lysates are approximately 2 ng/ml in MultiPL'100 and 3.5 ng/ml in MultiPL'30 (Maco Pharma International GmbH, MultiPL'30 and MultiPL'100 information sheets; 2017). The concentration of EGF in noncommercially available platelet lysates varies between 0.01 ng/ml and 18.3 ng/ml, depending on the donor and the manufacturing process [17, 31, 35]. Since we used 10% hPL and 20% FCS for medium supplementation, the EGF concentration in the cell culture media supplemented with MultiPL'30 and MultiPL'100 was half the concentration available in media supplemented with FCS. Thus, the concentration of EGF in cell media was irrelevant with respect to HUVEC growth and proliferation.

Cell apoptosis and necrosis of HUVEC were enhanced but not significantly increased when the cell growth medium was supplemented with the three distinct hPLs, in concentrations of 8% each. TGF-*β*1 induces apoptosis via mitogen-activated protein kinases in endothelial cells [42]. The concentration of TGF-*β*1 is significantly higher in hPLs (up to 769 ng/ml) than in FCS (up to 6.2 ng/ml) [17, 20, 35]. Thus, an increased rate of apoptosis was expected for cells treated with hPLs. Furthermore, IGF-1 plays a key role in the prevention of apoptosis [43]. The concentration of IGF-1 is up to 73 ng/ml in FCS [17, 20]. The concentration of IGF-1 is 45 ng/ml in MultiPL'30 and 70 ng/ml in MultiPL'100 (Maco Pharma International GmbH, MultiPL'30 and MultiPL'100 information sheets; 2017). In noncommercially available hPLs, the IGF-1 concentration varies from 15 ng/ml to 109 ng/ml [17, 20, 35]. This could explain the increased apoptosis rate in cells grown in media supplemented with 20% FCS or with 10% hPL. Moreover, necrosis is triggered by cellular stress. The switch of medium supplemented with different lysates three days prior to the experiments could trigger cellular necrosis. The enhanced apoptosis and necrosis rate could also be related to the high seeding density of cells. Nevertheless, the necrosis and apoptosis rate in HUVEC cultivated with medium supplemented with hPLs was not significantly higher than that in cells cultivated in medium supplemented with FCS. Thus, the composition and concentration of growth factors contained in hPLs did not negatively impair cell apoptosis and necrosis. Since the overall cell viability within the culture was not significantly affected, the documented increase in cell apoptosis and cell necrosis appeared to be compensated by simultaneously occurring increased proliferation.

The ability of endothelial cells to form tubes in 2D-cell culture provides information on their ability to reorganize for angiogenesis and form capillary-like structures during 3D-cell culture. Previous studies showed that thermosensitive hydroxybutyl chitosan hydrogels supplemented with 10% hPL promoted tube formation of HUVEC [44]. In the present study, the formation of tubes by HUVEC was improved when cell culture media were supplemented with hPLs compared to the cells cultured in media supplemented with FCS. The most promising hPL concentration for tube formation was a medium supplemented with either 5% MultiPL'30 or 8% MultiPL'100 or 10% lp-hPL. This indicated that using media supplemented with hPLs offered clear advantages in the formation of capillary-like structures compared to the use of media supplemented with FCS, especially when using standardized, commercially available hPLs. We hypothesize that this effect is mediated by VEGF, a protein which is crucial for angiogenesis [5, 45]. As described previously, the concentrations of VEGF in hPLs are much higher than in FCS [17, 20]. In addition, as described above, MultiPL'30 contains a higher concentration of VEGF than MultiPL'100. In both cases, the concentration is standardized between different batches. This might explain why HUVEC formed more pronounced tubes when cultivated in media supplemented with hPLs, even if the concentrations of the lysates were lower, compared to cells grown in media supplemented with FCS and why MultiPL'30 delivered the best results.

This study demonstrated that there were differences between the commercially available hPLs and laboratory-prepared lysates. The replacement of FCS with lp-hPL led to a recognizable altered morphology of the cells in a concentration-dependent manner. HUVEC showed an irregular shape and increased vacuolization of the cytoplasm, presumably due to cellular stress [46]. This was not the case when using MultiPL'30 and MultiPL'100. Moreover, an adequate tube formation was achieved only when the medium was supplemented with 10% lp-hPL. In contrast, adequate tube formation was achieved with lower concentrations of MultiPL'100 (8%) and MultiPL'30 (5%). On one hand, the noncommercial hPL has the advantage of being obtained by autologous methods; on the other, it may display large variations within the concentrations of growth factors [17]. In contrast, the growth factor concentrations of commercially available platelet lysates are standardized and vary only minimally from batch to batch. In addition, the patient's medical history must be considered when preparing autologous lysates. To date, oncological safety data regarding the use of autologous platelet lysates in tumor patients are lacking. As a result, there is a clear advantage for the use of standardized, commercially available hPLs for the replacement of FCS in cell culture media. Commercially available hPLs offer a safe and reproducible alternative to FCS for patients in need for tissue-engineered products. Since angiogenesis is of great importance for tissue engineering applications, this study indicates that the use of MultiPL'30 is the most suitable substitute for FCS for the cultivation of primary HUVEC in order to obtain prevascular structures.

In this study, we showed that hPLs are an adequate substitute for FCS as supplement in cell growth media required for *in vitro* cultivation of HUVEC, which showed no distinct donor-dependent variation in the results. Human platelet lysates in concentrations of 8% to 10% exhibited equal or higher effects regarding the formation of capillary-like structures as compared to media containing 20% FCS. Since angiogenesis is of great importance for tissue engineering purposes, the commercially available MultiPL'30 proved to be the most suitable supplement for the culture of primary HUVEC in order to obtain tube-like structures. With this study, we narrowed the gap between bench and bedside, by proposing a safe and efficient alternative for clinical implementation of in vitro engineered tissue grafts. This is a major step towards the engineering of prevascularized tissues for clinical applications.

## Data Availability

All relevant data are included within the manuscript.
